# GDF15 Supports the Inflammatory Response of PdL Fibroblasts Stimulated by *P. gingivalis* LPS and Concurrent Compression

**DOI:** 10.3390/ijms222413608

**Published:** 2021-12-19

**Authors:** Albert Stemmler, Judit Symmank, Julia Steinmetz, Katrin von Brandenstein, Christoph-Ludwig Hennig, Collin Jacobs

**Affiliations:** Department of Orthodontics, University Hospital Jena, Leutragraben 3, 07743 Jena, Germany; albert.stemmler@uni-jena.de (A.S.); julia.steinmetz@uni-jena.de (J.S.); Katrin.Brandenstein@med.uni-jena.de (K.v.B.); Christoph-Ludwig.Hennig@med.uni-jena.de (C.-L.H.); jacobscollin@googlemail.com (C.J.)

**Keywords:** periodontal disease, GDF15, orthodontic tooth movement, inflammation, PdL

## Abstract

Periodontitis is characterized by bacterially induced inflammatory destruction of periodontal tissue. This also affects fibroblasts of the human periodontal ligaments (HPdLF), which play a coordinating role in force-induced tissue and alveolar bone remodeling. Excessive inflammation in the oral tissues has been observed with simultaneous stimulation by pathogens and mechanical forces. Recently, elevated levels of growth differentiation factor 15 (GDF15), an immuno-modulatory member of the transforming growth factor (TGFB) superfamily, were detected under periodontitis-like conditions and in force-stressed PdL cells. In view of the pleiotropic effects of GDF15 in various tissues, this study aims to investigate the role of GDF15 in *P. gingivalis*-related inflammation of HPdLF and its effect on the excessive inflammatory response to concurrent compressive stress. To this end, the expression and secretion of cytokines (IL6, IL8, COX2/PGE2, TNFα) and the activation of THP1 monocytic cells were analyzed in *GDF15* siRNA-treated HPdLF stimulated with *P. gingivalis* lipopolysaccharides alone and in combination with compressive force. *GDF15* knockdown significantly reduced cytokine levels and THP1 activation in LPS-stimulated HPdLF, which was less pronounced with additional compressive stress. Overall, our data suggest a pro-inflammatory role for GDF15 in periodontal disease and demonstrate that GDF15 partially modulates the force-induced excessive inflammatory response of PdLF under these conditions.

## 1. Introduction

Growth differentiation factor 15 (GDF15), which belongs to the transforming growth factor (TGFB) superfamily [1], is strongly associated with several diseases such as obesity, cancer and cardiovascular diseases as well as with aging [2,3,4]. Under physiological conditions, GDF15 is only weakly expressed in most tissues, whereas pathological states and cellular stress strongly increase GDF15 levels [5,6,7], suggesting it as potential biomarker. Contributing to the regulation of cell differentiation and inflammation, GDF15 has also been shown to be involved in the regulation of cell repair and cell death [8]. Similar to several TGF-β family members [9], several studies also reported the role of GDF15 in the regulation of bone metabolism-related processes [10,11,12]. We have recently demonstrated that GDF15 is expressed and secreted by human periodontal ligament fibroblast (HPdLF) in a force-dependent manner [13], which is also supported by the studies of Li et al. [14].

The tissue of the periodontal ligament consists of a remarkably heterogeneous cell population, including osteoblast, osteoclasts, cementoblasts, epithelial rests of Malassez cells, macrophages, endothelial cells, neural cells, stem cells, and fibroblast [15]. The multipotent properties of adult PdL stem cells make them particularly interesting for potential treatment concepts for a wide variety of diseases [16]. With relatively modest effort, they can be isolated, cultured and differentiated into neural, mesodermal, osteoblast/cementoblast-like cells, adipocytes and chondrogenic cells by the addition of various factors [16,17]. The differentiation potential of PdL fibroblasts is mainly limited to the osteogenic lineage [18]. However, as the predominant cell type of the periodontal ligament they play an important role in maintaining periodontal health and functionality [19,20]. Located between the teeth and the alveolar bone, PdLF modulate force and pathogenic-induced local synthesis and release of various key inflammatory molecules such as prostaglandin E2 (PGE2), various interleukins (IL1, IL6 and IL8) as well as TNFα [20,21,22].

Mechanical forces occur especially during orthodontic interventions to achieve tooth movement but also during traumata. For tooth movement, transient aseptic inflammation is necessary for tissue and bone remodeling, as it contributes to the activation of osteoclasts and osteoblasts, which are responsible for bone resorption and new bone formation [19,22], respectively. Thus, dysregulated inflammatory processes, as in patients with periodontitis, can pose a great risk to the outcome of orthodontic treatment and potentially lead to tooth root degradation or even tooth loss [23,24]. Conversely, the correction of malocclusions can have a positive effect on oral health and thus reduce the risk of periodontal disease [25].

Periodontal inflammation, a complex, infectious, oral disease is characterized by bacterial-induced inflammatory destruction of tooth-supporting tissues [26]. Current concepts suggest a “polymicrobial synergy and dysbiosis” model that emphasizes the ability of keystone pathogens to modulate host response and thus significantly affect synergistic balance [27]. *Porphyromonas gingivalis* (*P. gingivalis*), one of the bacteria thought to be pathogenic in periodontal disease, has been studied extensively due to its unique ability to evade the immune response [28]. Thus, it impairs innate immunity to alter the growth and development of the entire biofilm and may trigger a destructive change in the normal homeostatic interaction between host and microorganisms in the subgingival plaque [29]. Typical virulence factors that lead to periodontal tissue damage include extracellular proteases, fimbria, and gingipain as well as lipopolysaccharides (LPS). Although both *P. gingivalis* and its LPS cannot cause periodontitis alone, in vitro cultivation with *P. gingivalis* LPS has been shown to stimulate the production of pro-inflammatory cytokine and is, therefore, used to mimic periodontitis-causing conditions.

Studies in mice suffering from polymicrobial infection including *P. gingivalis* revealed increased expression of *Gdf15* in PdL tissue [30]. However, whether GDF15 contributes to the modulation of the inflammatory response of PdL fibroblasts to *P. gingivalis* infection remains unknown. Moreover, to our knowledge, no study has yet investigated the role of GDF15 in the enhanced inflammatory response of force-stimulated HPdLF that typically occurs with concurrent *P. gingivalis*-LPS infection [31,32]. Therefore, the aim of this study was (1) to investigate the role of GDF15 in the inflammatory response of HPdLF to *P. gingivalis*-LPS in vitro and (2) to reveal GDF15-dependent changes when these cells are additionally loaded with compressive force.

## 2. Results

### 2.1. GDF15 Is Involved in the Inflammatory Response to P. gingivalis LPS

**Figure 1:**
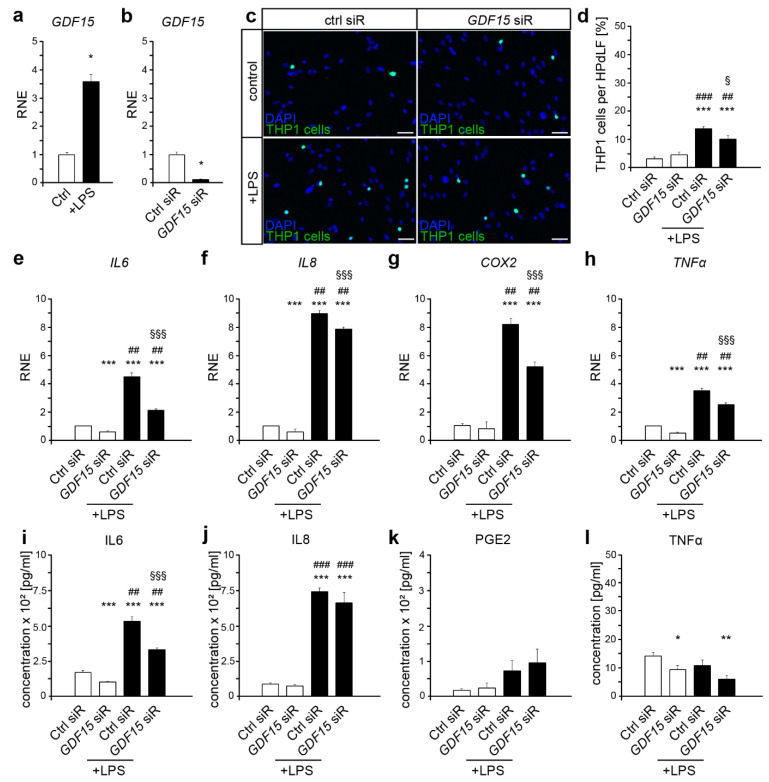
GDF15 promote the inflammatory response of HPdLF to lipopolysaccharides of *P. gingivalis*. (**a**) Quantitative analysis of *GDF15* expression in HPdLF stimulated with 10 µg/ml *P. gingivalis* LPS for 24 hours. (**b**) Validation of *GDF15* knockdown by quantitative analysis of *GDF15* expression level in HPdLF treated with *GDF15* siRNA (*GDF15* siR) or Control siRNA (Ctrl siR). (**c**,**d**) Analysis of adherent THP1 monocytic cells (green, Alexa488-gelabled) on HPdLF (blue, DAPI) after *GDF15* knockdown as well as stimulation with *P. gingivalis* LPS (**c**). The relative number of THP1 cells is displayed per 100 HPdLF in (**d**). (**e**–**h**) Quantitative expression analysis of the inflammatory genes *IL6* (**e**), *IL8* (**f**), *COX2* (**g**) and *TNFα* (**h**) in *GDF15*-deficient HPdLF additionally stimulated with *P. gingivalis* LPS. (**i**–**l**) Analysis of secreted cytokines IL6 (**i**), IL8 (**j**), PGE2 (**k**) and TNFα (**l**) in *GDF15*-deficient HPdLF additionally stimulated with *P. gingivalis* LPS. * *p* < 0.05; ** *p*  <  0.01; *** *p* < 0.001 in relation to Ctrl (**a**) and Ctrl siR (**b**–**l**), ^##^ *p* < 0.01; ^###^ *p* < 0.001 in relation to GDF15 siR, § *p* < 0.05; §§§ *p* < 0.001 in relation to Ctrl siR + LPS; One-Way ANOVA and post hoc test (Tukey). Scale bars: 50 μm in (**c**). RNE, relative normalized expression.

*P. gingivalis*-LPS induce a strong pro-inflammatory response of HPdLF [21,31,32]. We could detect an increased expression of *GDF15* in six hours LPS-stimulated HPdLF (Figure 1a). To elucidate the role of GDF15 in the response of HPdLF to the pathogenic stimulus, we performed siRNA-induced *GDF15* knockdown (Figure 1b). *GDF15* deficiency did not alter the activation of monocytes as shown by the THP1 adhesion assay (Figure 1c,d). However, the increase in adherent THP1 cells due to LPS treatment was significantly lower in GDF15-deficient cells compared with control siRNA-treated HPdLF, indicating an important role of GDF15 in inflammatory response to pathogenic stimuli.

To further analyze GDF15-dependent changes in inflammatory signaling mediators that are important for THP1 activation, we performed qPCR of *IL6*, *IL8*, *COX* and *TNFα* in treated HPdLF, respectively (Figure 1f). LPS stimulation induced an upregulated expression of all cytokines in control siRNA-treated HPdLF, which was significantly lower upon *GDF15* knockdown. Remarkably, *GDF15*-deficient cells also showed reduced expression of *IL6*, *IL8* and *TNFα* when not stimulated with LPS. However, transcript analysis suggested a pro-inflammatory role of GDF15 in pathogenic response of HPdLF. To validate RNA data, we analyzed cytokine secretion in the supernatant of stimulated cells, respectively (Figure 1i–l). Although PGE2 and TNFα secretion remained unchanged, LPS stimulation resulted in increased IL6 and IL8 levels in control as well as *GDF15* siRNA-treated HPdLF. Although we did not detect GDF15-dependent changes in IL8 secretion, the increase in IL6 was significantly reduced in *GDF15*-deficient HPdLF compared with the respective controls. Altogether, our data suggest a pro-inflammatory role of GDF15 in the response of HPdLF to pathogenic stimuli, which seems to depend on IL6 signaling.

### 2.2. GDF15 Promotes the Inflammatory Response of HPdLF to Compressive Force

**Figure 2:**
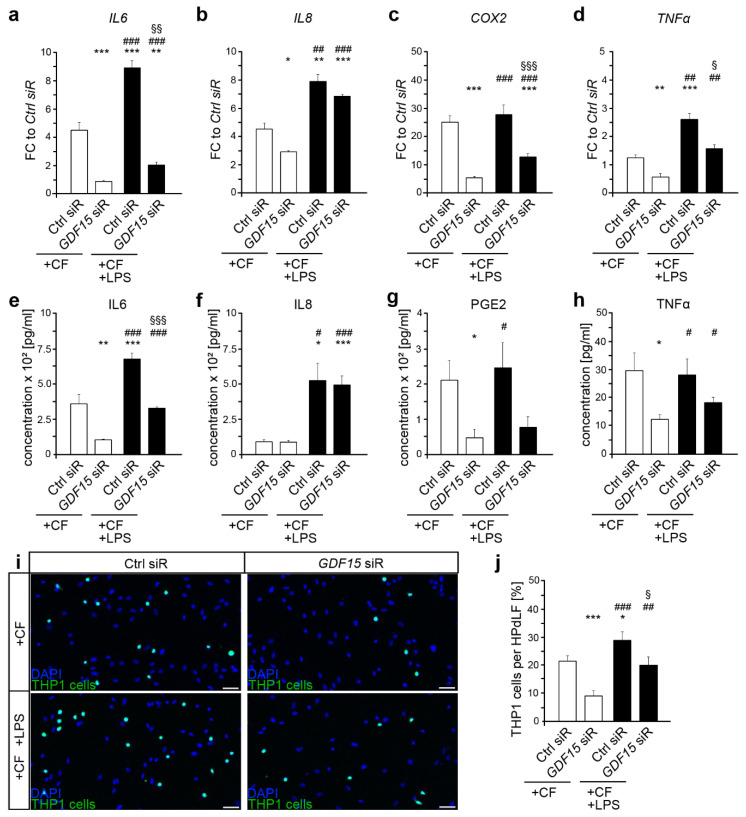
The pro-inflammatory response of HPdLF to mechanical and bacterial stimuli is partially modulated by GDF15. (**a**–**d**) Quantitative expression analysis of the inflammatory genes *IL6* (**a**), *IL8* (**b**), *COX2* (**c**) and *TNFα* (**d**) in HPdLF treated with *GDF15* siRNA (*GDF15* siR) or Control siRNA (Ctrl siR), stimulated for six h with compressive force (+CF, white bars) and lipopolysaccharides of *P. gingivalis* (+LPS, black bars). The expression levels are displayed as fold change (FC) to Ctrl siR-treated cells. (**e**–**h**) Analysis of secreted cytokines IL6 (**e**), IL8 (**f**), PGE2 (**g**), and TNFα (**h**) in HPdLF under previous conditions. (**i**,**j**) Analysis of adherent THP1 monocytic cells (green, Alexa488-gelabled) on mechanically stressed *GDF15*-deficient HPdLF (blue, DAPI) in relation to the control under previous conditions (**i**). The relative number of THP1 cells is displayed per 100 HPdLF in (**j**). * *p* < 0.05; ** *p* < 0.01; *** *p* < 0.001 in relation to Ctrl siR +CF; ^#^ *p* < 0.05; ^##^ *p* < 0.01; ^###^ *p* < 0.001 in relation to GDF15 siR +CF, § *p* < 0.05; §§ *p* < 0.01; §§§ *p* < 0.001 in relation to Ctrl siR +CF +LPS. One-Way ANOVA and post hoc test (Tukey). Scale bars: 50 μm in (**c**).

Compressive force, as it occurs during orthodontic treatments, not only induces a release of pro-inflammatory cytokines [20,21,22], but also of GDF15 [13]. With the exception of *TNFα*, six hours of mechanical compression led to an increase in the expression levels of all cytokines analyzed (Figure 2a–d white bars, compared with Figure 1e–h white bars; *IL6* *p*-value = 0.014 × 10^−2^, ***; *IL8* *p*-value = 0.045 × 10^−2^, ***; *COX2* *p*-value = 0.003 × 10^−3^, ***; *TNFα* *p*-value = 0.875). In contrast to control siRNA-treated cells, *GDF15*-deficient HPdLF showed significantly reduced upregulation of *IL6*, *IL8* and *COX2* (Figure 2a–c, white bars). It should be mentioned that the *GDF15* knockdown in mechanically stressed HPdLF also resulted in a decrease in *TNFα* levels compared with force-stimulated siRNA-treated cells (Figure 2d, white bars). RNA expression data emphasize a pro-inflammatory role of GDF15 in the response of HPdLF to compressive stress, which is supported by cytokine secretion analysis. Six-hour compressive force triggered increased secretion of IL6 and PGE2, but not of IL8 and TNFa, in control siRNA-treated cells (Figure 2e–h white bars, compared with Figure 1e–h; IL6 *p*-value = 0.013, *; IL8 *p*-value = 0.980; PGE2 *p*-value = 0.004, **; TNFα *p*-value = 0.072), levels of these cytokines were significantly lower in force-stressed *GDF15*-deficient HPdLF (Figure 2e–h, white bars). Moreover, THP1 assay confirmed the pro-inflammatory function of GDF15 as gene knockdown resulted in a lower number of adherent THP1 cells (Figure 2i,j white bars). In agreement with data of Li et al. [14], GDF15 seems to promote the pro-inflammatory response of PdL cells to compressive stimuli. 

### 2.3. Additional Exposure to Bacterial Stimulants Enhanced the Inflammatory Response in Mechanically Stressed HPdLF, Even in the Presence of GDF15 Deficiency

Because GDF15 appears to be involved in modulating the inflammatory response to both, pathogenic and mechanical stimuli, we were interested in its role in a concurrent exposure. Although *COX2* expression was not affected, simultaneous exposition to pathogenic and mechanical stimuli led to even higher expression levels of *IL6*, *IL8* and *TNFα* in control siRNA-treated HPdLF compared with compressed controls that were not stimulated with LPS (Figure 2a–d). When comparing *GDF15*-deficient HPdLF that were mechanically and bacterially stressed with those that were not additionally stimulated with LPS, dual stimulation resulted in an increase in the expression of all cytokines analyzed. However, with the exception of IL8, the expression levels were still lower than those of siRNA-treated HPdLF (Figure 2a–d black bars). It should be noted that compared with respective LPS-stimulated cells, excessive expression of *IL6* and *COX2* was detected in concurrently stimulated controls, whereas only *COX2* levels were upregulated in *GDF15*-deficient HPdLF (Figure 2a–d black bars, compared with Figure 1e–h black bars; control siRNA *IL6 p*-value = 0.001 × 10^−2^, ***; *IL8 p*-value = 0.856; *COX2 p*-value = 0.002 × 10^−3^, ***; *TNFα p*-value = 0.785; *GDF15* siRNA *IL6 p*-value = 0.985; *IL8 p*-value = 0.891; *COX2 p*-value = 0.001 × 10^−2^, ***; *TNFα p*-value = 0.698).

Analysis of cytokine secretion confirmed the increase in IL6 and IL8 levels for control siRNA and GDF15 siRNA-treated HPdLF concomitantly exposed to mechanical and pathogenic stimuli compared with cells that were not stimulated with LPS, whereas PGE2 levels remained unaffected. (Figure 2e–g). However, although IL8 secretion was comparatively high, IL6 levels were lower in HPdLF with reduced GDF15 expression supporting RNA expression data (Figure 2e,f black bars). Furthermore, and in contrast to the control, *GDF15*-deficient HPdLF also showed slightly increased TNFα secretion when stimulated with *P. gingivalis* LPS in addition to mechanical compression (Figure 2h). Compared with LPS-stimulated HPdLF, control siRNA-treated HPdLF showed a further increase in cytokine secretion due to concurrent stimulation for IL6, PGE2, and TNFα, whereas GDF15-deficient cells showed only an upregulation of TNFα secretion only (Figure 2e–h black bars, compared with Figure 1e–h black bars; control siRNA IL6 *p*-value = 0.014, *; IL8 *p*-value = 0.133; PGE2 *p*-value = 0.042, *; TNFα *p*-value = 0.020; *GDF15* siRNA IL6 *p*-value = 0.714; IL8 *p*-value = 0.129; PGE2 *p*-value = 0.730; TNFα *p*-value = 0.045 × 10^−2^, ***).

To find out to what extent these changes in cytokine secretion affects the adhesion of THP1 cells, we performed the corresponding assay (Figure 2i,j). Both control siRNA and *GDF15* siRNA-treated HPdLF showed increased adhesion of THP1 cells, indicating an excessive inflammatory response when the cells were stimulated with *P. gingivalis* LPS in addition to mechanical compression. However, the number of adherent THP1 cells was only slightly decreased in *GDF15* deficiency (Figure 2i,j black bars), suggesting that in dual stimulation with mechanical and pathogenic stimuli, other key factors besides GDF15 play a modulatory role in the inflammatory response of HPdLF. This was also confirmed by comparing the adhesion values of dual-stimulated HPdLF with LPS-stimulated only. Although LPS-induced increase in adhesive THP1 cells resulted in a significant different fold changes of 7.73 ± 1.54 in control siRNA-treated HPdLF versus 3.76 ± 0.86 in GDF15-deficient HPdLF (*p*-value = 0.033, *), additional mechanical stimulation revealed comparable fold changes of 2.07 ± 0.18 in control versus 2.76 ± 0.58 in GDF15-deficient cells (*p*-value = 0.273).

Taken together, our data indicate that GDF15 is highly important for the pro-inflammatory response of HPdLF to *P. gingivalis* LPS and mechanical compression. However, with simultaneous exposure to both stimuli, additional mechanisms and regulators appear to act independently of GDF15. 

## 3. Discussion

This study indicates a pro-inflammatory role of GDF15 in pathogenic and mechanical stimuli of human periodontal ligament fibroblasts associated with periodontitis and orthodontic tooth movement. Knockdown of *GDF15* resulted in reduced release of pro-inflammatory cytokines and a lower number of activated monocytic cells after pathogenic stimulation with *P. gingivalis* LPS as well as after six hours of mechanical compression. Thus, GDF15 seem to be involved in the modulation of the inflammatory response of these cells. However, concurrent stimulation, which triggers a severely increased inflammatory response also appears to activate signaling pathways or regulators that are independent of GDF15.

There is growing evidence for the involment of GDF15 in disease and inflammation. Contextually GDF15 appears to have a pleiotropic role, as both pro- and anti-inflammatory effects have been described. With regard to periodontistis, an increased expression of *Gdf15* was recently detected in BALB/cByJ mice after polymicrobial oral inoculum with *P. gingivalis*, *Treponema denticola*, *Tannerella forsythia*, and *Fusobacterium nucleatum* [30]. We have now shown that in vitro the stimulation with *P. gingivalis* LPS alone is sufficient for an increase in *GDF15* expression in human PdL fibroblasts. *P. gingivalis* LPS has been shown to activate *Toll-like-receptor* 4 (TLR4), but not by TLR2 [33]. However, this appears to be cell-type-specific, as TLR2 activation has also been reported [34]. In this regard, it has been reported that an activated TLR2-Myd88 pathway simulates the secretion of GDF15 [35], and may act as downstream signaling mediator. 

Our data revealed that GDF15 act on specific cytokines in HPdLF, as mainly decreased IL6 secretion levels was detected by LPS stimulation in *GDF15*-deficient cells, whereas IL8 increase was GDF15-independent. This appears to be partly consistent with studies in human nasal epithelial cells, where knockdown reduced the LPS-mediated expression and secretion of IL6, but also of IL8 and TNFα. Moreover, upregulation of GDF15 led to a marked increase in inflammation in the expression and secretion of inflammatory cytokines in these cells [36]. According to this, overproduction of GDF15 also seems to promote human rhinovirus (HRV) infection and pathogen-induced inflammation in lung [37]. In this context, HRV-induced IL6, KC and IP10 protein levels were significantly higher in hGDF15 Tg+ mice compared with wild-type littermates [37]. However, the functionality of GDF15 seems to depend on a variety of conditions, including the organism, surrounding tissue, age, and stimuli. Thus, other studies reported that GDF15 seems to have tissue protective effects in heart, liver, kidney and lung by reducing the extent of damage after injury and inflammation following these events [38,39,40,41]. Abulizi et al. [42] demonstrated that a lack of GDF15 upregulates the expression of inflammatory cytokines induced by LPS stimulation. In comparison with wild-type or GDF15 transgenic mice, GDF15 knockout mice treated with LPS expressed significantly higher levels of MCP1, KC, IL6 and TNFα. [42]. However, it was also shown that pretreatment with GDF15 did not inhibit LPS-stimulated expression of inflammatory cytokines in the mouse macrophage cell line RAW 264.7, mouse peritoneal macrophages and mouse liver Kupffer cells [43].

In addition to its apparently important role in modulating the inflammatory response to pathogenic stimuli, GDF15 has several regulatory functions in the response to compressive forces. For instance, in pancreatic as well as in brain cancer cells it was recently shown that tumor-dependent compressive cellular stress upregulates cell migration in a GDF15 manner [44,45]. The function of GDF15 in mechanically induced inflammatory processes of the PdL was recently investigated by Li et al. [14] using isolated PDL cells from extracted human premolars loaded with a static compressive force of 1.5 g/cm^2^ for 12 hours. Similar to this study, we found decreased expression of *IL6*, *IL8* and *COX2* in *GDF15*-deficient cells at six hours of static compressive force of 2 g/cm^2^ supporting the pro-inflammatory role of GDF15 in mechanically induced PdL inflammation. Moreover, our study now provides evidence that GDF15 also modulates TNFα levels in this context. Even if the secretion of the cytokine did not increase under the conditions used, GDF15 deficiency leds to downregulation of baseline TNFa levels in non-compressed cells close to the lower detection limit of the ELISA assay. In this context, Bootcov et al. [1] reveals for the first time that GDF15 is able to significantly inhibit TNFa secretion in macrophages stimulated by LPS. Accordingly, suppressed TNFa and iNOS gene expression was observed in RAW264.7 macrophages after treatment with recombinant GDF15 [46]. In the past, it has been reported that anti-inflammatory cytokines such as IL4, IL10 and IL13 regulate TNFa activity [47,48,49,50,51]. Nevertheless, data on TNFa regulation by cytokines, including GDF15, are limited so far, which may provide avenues for further investigation.

Several studies suggest that TLR’s play a crucial role not only in pathogen defense, but also in the intracellular transmission of mechanosensitive stimuli. In this regard, it has not only been shown that orthodontic forces promote the expression of TLR2 and TLR4 in PdL cells, but also that these receptors are important for the inflammatory response to mechanical stress [52,53]. This immunomodulatory effect of mechanical forces could explain the excessive pro-inflammatory response to additional pathogenic stimuli by HPdLF. In particular, increased expression and secretion of IL8 and PGE2 was detected upon simultaneous bacterial and mechanical stimulation of PdL fibroblasts [21,54]. Here, we observed an increase in PGE2, but also in IL6 and TNFα, whereas IL8 protein levels remained unchanged by dual stimulation. These differences from other studies could be due to differences in LPS composition, amount and duration of stimulation, and strength and duration of mechanical force application. Furthermore, posttranscriptional regulation of IL8 levels was found through an increase in PGE2 [21]. In any case, GDF15 seems to be relevant for the expression/secretion of these cytokines, with the exception of IL8, but is not exclusively responsible for excessive inflammation in the context of concurrent stimulation since, in addition to the cytokines studied, a variety of other pro-inflammatory signaling mediators such as IL1α, IL1β, IL1RA, IFNγ, etc., are crucial for the inflammatory response of HPdLF, this is not so improbable. However, this indicates the limitations of our study. Further intensive cyokine studies could reveal a complete image of GDF15-dependent and -independent cytokines in these context of stimulation, which was, however, beyond the scope of our study.

It should be noted that periodontitis is considered as multifactorial triggered disease, and isolated LPS stimulation by *P. gingivalis* can only partially simulate the pathogenesis. In this regard it is also important to mention that the extent of periodontal inflammation depends not only on the bacterial infection, but also on the host-specific inflammatory response, which in vivo may be influenced by certain preexisting health conditions as well as by ageing. In this context, our study is limited to the in vitro effects of isolated lipopolysaccharides, which represent only a simplified model of the in vivo situation of periodontal disease. Moreover, our studies have focused only on the modulating effects of GDF15 under compressive loading. Since both compressive and tensile forces contribute significantly in vivo to tissue as well as bone remodeling, and finally tooth movement, future studies could focus on the function of GDF15 in stretched PdL fibroblasts. In addition, compression was limited to 6 hours in our experimental design, which is still quite low for triggering a robust inflammatory response. Variations could provide important clues to the different phases of orthodontic tooth movement, as well as a more complete understanding of the underlying molecular mechanism.

Nevertheless, our study offers a first insight into how GDF15 regulates the inflammatory response of PdL fibroblasts caused by periodontal pathogens and what impact this might have on additional orthodontic treatment. Our study was a first important contribution to further understanding of the modulatory potential of GDF15, which is still poorly understood in this context. Therefore, we demonstrate a pro-inflammatory role of GDF15 in the response of human periodontal ligament fibroblasts to pathogenic and mechanical stimuli by modulating the expression and secretion of key cytokines and the activation of monocytic cells. However, our data are based on in vitro approaches and should be validated in vivo. Blocking GDF15 signaling may provide a potential opportunity to limit the excessive inflammatory response in PdLF of orthodontic patients suffering from periodontal disease. For this purpose, the use of inhibitory antibodies targeting GDF15 or the GDF15-specific receptor GFRAL are the current primary options [55,56]. However, GFRAL is expressed in a neuron-specific manner, which precludes the possibility of its inhibition in PdL fibroblasts [57]. In this regard, activin receptor-like kinase 5 (ALK5), which is also expressed in PdLF [58], was recently suggested as another GDF15 receptor [59]. Hence, in order to achieve more comprehensive understanding and possible expansion of diagnostic and therapeutic treatment options associated with efficient orthodontic tooth movement in pathological conditions, further research of GDF15 signaling and function is essential.

## 4. Materials and Methods

### 4.1. Cell Culture

Pooled human periodontal ligament fibroblasts from several donors (HPdLF, Lonza, Basel, Switzerland) were grown in culture medium consisting of Dulbecco’s modified Eagle medium (DMEM; Thermo Fisher Scientific, Carlsbad, CA, USA) containing 4.5 g/L glucose, 10% heat-inactivated fetal bovine serum (Thermo Fisher Scientific, Carlsbad, CA, USA), 100 U/mL penicillin, 100 µg/mL streptomycin and 50 mg/L L-ascorbic acid at 37 °C, 5 % CO_2_ and 95% humidity. Cells were regularly passaged with 0.05% Trypsin/EDTA (Thermo Fisher Scientific, Carlsbad, CA, USA) when 75% confluence was reached. HPdLF of passage four to eight were used for experimental setups. For RNA and protein expression analysis, 1 × 10^5^ cells were seeded per well of a 6-well plate and cultured to 75% confluency prior further treatment. For THP1 cell adherence assay, 50 HPdLF per mm^2^ were seeded on coverslips in 24-well plates and cultured to 75% confluence.

THP1 monocytic cells (DMSZ, Braunschweig, Germany) were cultured in RPMI 1640 medium (Gibco) containing 10 % FBS, 100 U/mL penicillin and 100 µg/mL streptomycin at 37 °C, 5 % CO_2_ and 95 % humidity. They were passaged weekly and seeded at a density of 1 × 10^6^ cells in 20 mL medium in T175 culture flask (Thermo Fisher Scientific, Carlsbad, CA, USA).

### 4.2. siRNA-Mediated Knockdown

For siRNA-targeted *GDF15* knockdown, siRNA transfection was performed with Lipofectamine^TM^ 2000 (Thermo Fisher Scientific, Carlsbad, CA, USA) according to the manufacturer’s protocol. Human *GDF15* siRNA oligos (50 nM, Santa Cruz Biotechnology, Dallas, TX, USA) or 50 nM BLOCK-iT Alexa Fluor red control siRNA (Thermo Fisher Scientific, Carlsbad, CA, USA) were applied for five hours in OptiMem I-reduced serum medium (Thermo Fisher Scientific, Carlsbad, CA, USA) containing 100 U/mL penicillin and 100 µg/mL streptomycin. Then, HPdLF were further grown in the culture medium. Transfection efficiency was analyzed by fluorescence microscopy and ranged from 93.5 to 98.7% for all conditions.

### 4.3. P. gingivalis LPS Stimulation

For pathogenic stimulation, 10 µg/mL lipopolysaccharides of *P. gingivalis* (InvivoGen, San Diego, CA, USA) were added to the culture medium for 24 h. LPS stimulation of siRNA-treated cells was performed immediately after siRNA-mediated knockdown in HPdLF culture medium. 

### 4.4. Mechanical Compression

The compressive force of 2 g/cm^2^ was applied based on the protocol of Kirschneck et al. [60] and as previously described [61]. Briefly, immediately after siRNA treatment and 24h after LPS application, glass plates were placed on the cells for six hours at 37 °C, 5% CO2 and 95% humidity. Cells were then either isolated directly with TRIzol reagent (Thermo Fisher Scientific, Carlsbad, CA, USA) for expression analysis or the medium was collected 24 h later for protein analysis. For the THP1 cell adherence assay, a compressive force of 7.13 g/cm^2^ was applied in 24-well plates by centrifugation at 30 °C for six h. These were the minimum conditions of the centrifuge. Control cells were cultured at 30 °C for the period of mechanical stimulation.

### 4.5. THP1 Cell Adherence Assay

To visualize inflammatory response of HPdLF, THP1 cell adhesion assay was performed as previously described [32]. Briefly, 50 × 10^3^ Celltracker CMFDA (Thermo Fisher Scientific, Carlsbad, CA, USA) stained non-adherent THP1 monocytic cells were added to each well of a 24-well plate with treated HPdLF. After cell adhesion for 30 min, non-adhered THP1 cells were removed by washing with prewarmed PBS. Coverslips were fixated in 4% paraformaldehyde for 10 min and washed in PBS, and nuclei were stained with DAPI (1:10000 in PBS) for 5 min. Coverslips were embedded with Mowiol® 4-88 (Carl Roth, Karlsruhe, Germany) on glass object slides for microscopic imaging. Each condition was analyzed at least in biological triplicates with technical duplicates per sample.

### 4.6. RNA Extraction and Quantitative PCR 

RNA expression analysis was performed as previously described [32]. Briefly, RNA was isolated with TRIzol Reagent (Thermo Fisher Scientific, Carlsbad, CA, USA)/1-bromo-3-chloropropane and purified with RNA Clean & Concentrator-5 kit (Zymo Research, Freiburg, Germany) according to the manufacture’s guidelines. RNA quantity and quality were tested with Nanodrop 2000 (Avantor, Radnor, PA, USA). cDNA synthesis was performed with SuperScript IV Reverse Transcriptase (Invitrogen) using Oligo(dt)_18_ primers (Thermo Fisher Scientific, Carlsbad, CA, USA) according to the manufacture’s protocol. Luminaris Color HiGreen qPCR Master Mix (Thermo Fisher Scientific, Carlsbad, CA, USA) was used for quantitative PCR, which was performed using qTOWER3 (Analytik Jena, Jena, Germany) according to the manufacturer’s guidelines. 

Primer design was performed as previously described [13]. Primer sequences for all genes studied are listed in Table 1, which also includes gene symbols, NCBI gene IDs, and lengths of forward and reverse primers. *RPL22* and *TBP* were used as reference genes. To evaluate the quality and specificity of the primers, melting curve analysis and agarose gel electrophoresis was performed. To calculate primer efficiency, dilution series of cDNA concentrations were tested. Data were analyzed with the efficiency-corrected ΔΔCT method [62]. Each condition was analyzed at least in biological triplicates with technical duplicates per sample.

### 4.7. Enzyme-Linked Immunosorbent Assay (ELISA)

To analyze cytokine seretion, IL6 (Quiagen, Hilden, Germany), IL8 (Quiagen, Hilden, Germany) and prostaglandin E2 (PGE2; R&D Systems, Minneapolis, MN, USA) ELISA were performed on the medium isolated from HPdLF according to the manufacturer’s guidelines. Each individual condition was tested at least in biological triplicates with technical duplicates per sample.

### 4.8. Microscopy, Image Analysis and Statistics

The inverted confocal laser scanning microscope TCS SP5 (Leica, Wetzlar, Germany) was used to image THP1 cell adherence assay. Fiji software (https://imagej.net/Fiji (accessed on 26 November 2021)) was used for cell number analysis. Graph Pad Prism (https://www.graphpad.com (accessed on 26 November 2021)) was used for statistical analysis and in addition to Adobe Photoshop CS5 (https://adobe.com (accessed on 26 November 2021)) for figure illustration. One-way ANOVA and a post hoc test (Tukey) were used as statistical tests. Significance levels: *p*-value < 0.05 *; *p*-value < 0.01 **; *p*-value < 0.001 ***.

## Figures and Tables

**Table 1:** qPCR primer sequences of human genes indicated in 5′-3′ direction. bp, base pairs. Length, amplicon length.

Gene	Gene Symbol	NCBI Gene ID	Primer Sequence	Length
growth differentiation factor 15	*GDF15*	9518	fw CCGAAGACTCCAGATTCCGA rew CCCGAGAGATACGCAGGTG	180 bp
C-X-C motif chemokine ligand 8	*IL8*	3576	fw TTGGCAGCCTTCCTGATTTCT rew GGTCCACTCTCAATCACTCTCA	149 bp
Interleukin 6	*IL6*	3569	fw CATCCTCGACGGCATCTCAG rew TCACCAGGCAAGTCTCCTCA	164 bp
Prostaglandin-endoperoxide synthase 2	*PTGS2* *(COX2)*	5743	fw GATGATTGCCCGACTCCCTT rew GGCCCTCGCTTATGATCTGT	185 bp
Ribosomal protein L22	*RPL22*	6146	fw TGATTGCACCCACCCTGTAG rev GGTTCCCAGCTTTTCCGTTC	98 bp
TATA-box binding protein	*TBP*	6908	fw CGGCTGTTTAACTTCGCTTCC rev TGGGTTATCTTCACACGCCAAG	86 bp
Tumor necrosis factor	*TNFα*	7124	fw CACGCTCTTCTGCCTGCTG rev AGGCTTGTCACTCGGGGTT	130 bp

## Data Availability

The datasets of this study are available from the corresponding author on reasonable request. The data are not publicly available due to very large size of microscopy images.
